# Supplementary figures and images for: Exploring the overlap between rheumatoid arthritis susceptibility loci and long non-coding RNA annotations
Source: PLoS One. 2020 Mar 20;15(3):e0223939. doi: 10.1371/journal.pone.0223939 (PMC7083320; doi:10.1371/journal.pone.0223939)

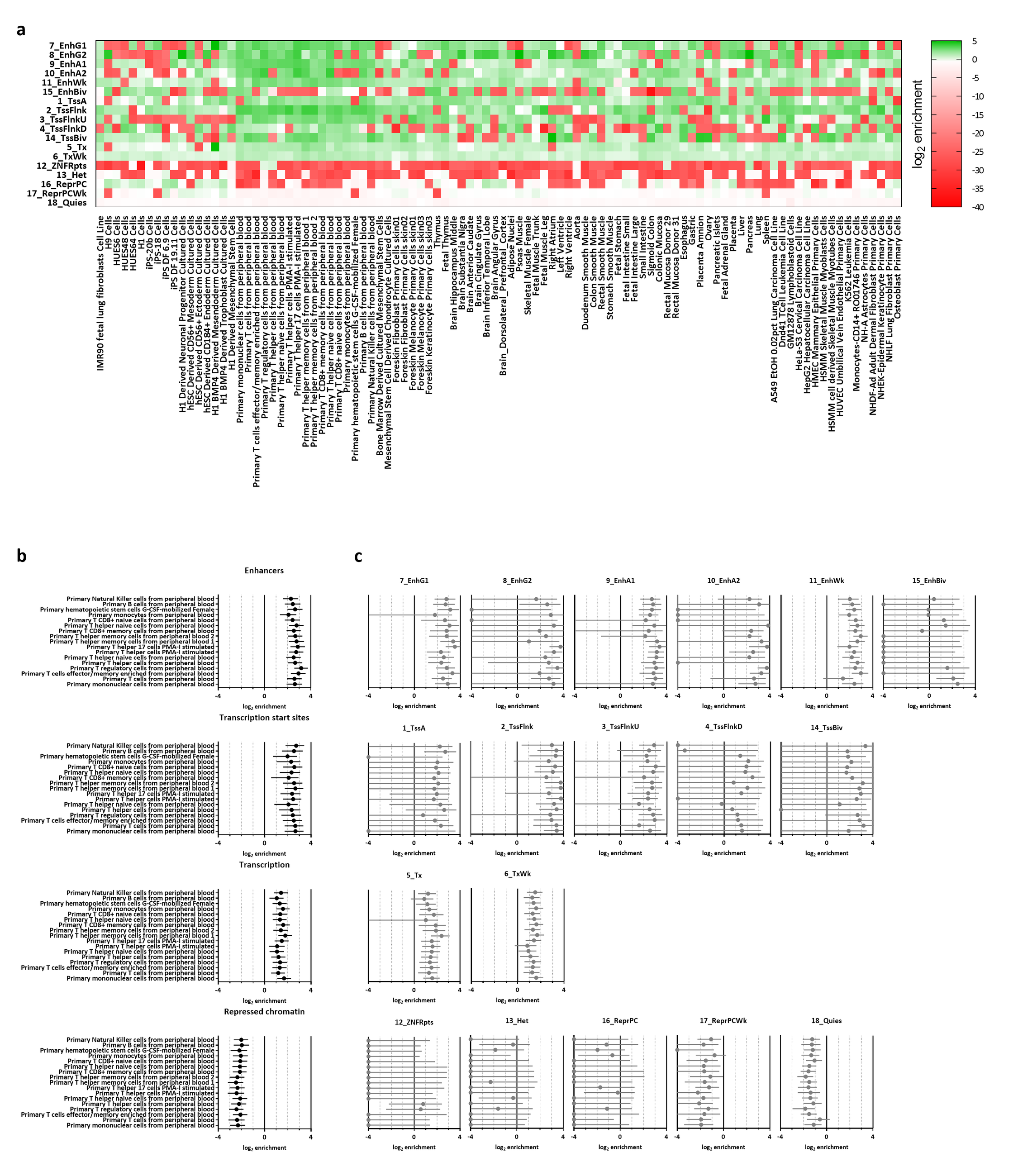

Supplement: S1 Fig — Estimates for enrichment of individual states are illustrated for 98 cell types using the Roadmap Epigenomics 18-state model (a). Similar states were grouped into four groups for all immune-relevant primary cell-types (b), as individual states often gave very broad 95% confidence intervals (c). Cell-types are ordered according to the clustering established by the Roadmap Epigenomics project, with chromatin states reordered according to their subsequent grouping. Estimates and confidence intervals are clipped at axis limits, where applicable. (TIF) [file pone.0223939.s001.tif]

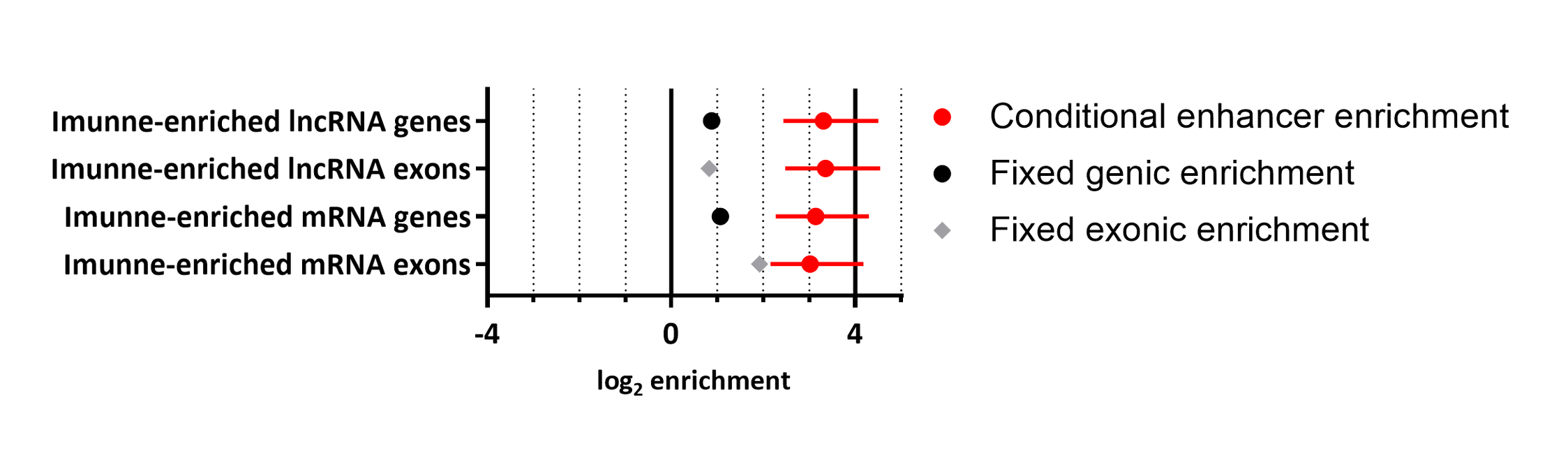

Supplement: S2 Fig — The influence of FANTOM CAT immune-enriched lncRNA and mRNA was fixed in a probabilistic model of RA susceptibility to confirm the independent enrichment of immune-relevant enhancer chromatin states. (TIF) [file pone.0223939.s002.tif]
